# Availability of integrated family planning services in HIV care and support sites in sub-Saharan Africa: a secondary analysis of national health facility surveys

**DOI:** 10.1186/s12978-019-0713-x

**Published:** 2019-05-29

**Authors:** Mufaro Kanyangarara, Kwame Sakyi, Amos Laar

**Affiliations:** 1 Department of International Health, Johns Hopkins Bloomberg School of Public Health, 615 N. Wolfe Street, Baltimore, MD USA; 2 Department of Public and Environmental Wellness, School of Health Sciences, Oakland University, Rochester, MI USA; 3 Department of Population, Family, and Reproductive Health, School of Public Health, College of Health Sciences, University of Ghana, Accra, Ghana

**Keywords:** HIV, Family planning, Sub-Saharan Africa, Health facility surveys

## Abstract

**Background:**

Integrating family planning (FP) with HIV care and treatment programs is a strategy to expand FP service delivery and prevent unintended pregnancies among women living with HIV. However, little is known about the extent to which FP services are available in health facilities providing HIV services across sub-Saharan Africa. In this study, we assessed the availability of integrated FP services and the associated factors in HIV care and support sites across sub-Saharan Africa.

**Methods:**

We conducted a secondary analysis of nationally representative facility-level data from Service Availability and Readiness Assessments (SARA) and Service Provision Assessments (SPA) conducted in 10 sub-Saharan African countries between 2012 and 2015. We used six indicators that reflect the structure and process of care essential for FP service delivery in HIV care and support facilities to define the outcome of interest - onsite availability of integrated FP services. Multivariate logistic regression was used to explore facility-level characteristics associated with the outcome.

**Results:**

Among the 3161 health facilities offering HIV care and support services, most reported also offering FP services at the same location. The availability of three FP methods was higher than the availability of FP guidelines and trained staff. Onsite availability of integrated FP services ranged from 10 to 61%. Results of multivariate logistic regression indicated that the odds of having onsite integrated FP services available was higher in HIV care and support sites that were operated by the government, classified as a tertiary level care facility, and provided services for PMTCT, antenatal care and basic surgery.

**Conclusions:**

Our findings indicate critical shortcomings in the preparedness of HIV care and support sites to deliver onsite integrated FP services. Renewed efforts are needed to address these supply-side barriers and ensure that integrated FP and HIV services meet the unique needs of HIV clients.

**Electronic supplementary material:**

The online version of this article (10.1186/s12978-019-0713-x) contains supplementary material, which is available to authorized users.

## Background

Since the start of the HIV epidemic, an estimated 78 million people have acquired HIV, and 35 million have died of AIDS-related causes [1]. In 2016, there were 36.7 million people living with HIV/AIDS worldwide, with 25.5 million residing in Sub-Saharan Africa. Annually about 2.1 million people become newly infected with HIV, including 150,000 children (< 15 years), most of whom acquire the infection vertically from their mothers, during pregnancy, childbirth or breastfeeding [1, 2].

The scale-up of interventions for the prevention of mother-to-child transmission of HIV (PMTCT) and antiretroviral treatment for eligible pregnant women and children has led to significant reductions in HIV transmission, morbidity and mortality [3]. The benefits associated with improved access to antiretroviral therapy (ART) may be offset by high rates of unintended pregnancies and low levels of contraceptive use among women living with HIV in sub-Saharan Africa [4–8]. Preventing unintended pregnancies in HIV-infected women is recognized an essential component of a comprehensive response to HIV/AIDS, especially the global PMTCT [9, 10]. The integration of family planning (FP) services into HIV/AIDS care and treatment programs is an effective approach to simultaneously reducing vertical transmission of HIV, increasing access to contraception, and reducing maternal deaths [11–13]. Furthermore, at a health systems level, the delivery of FP services as part of the HIV continuum of care can lead to improvements in access to essential health services, efficiency of limited resources and clinical practice [14–16]. Models to integrate HIV and FP service delivery range from the provision of comprehensive FP services from the same HIV service provider to referral-based approaches [17]. The effectiveness of a particular model of integration depends on contextual factors such as the burden of HIV and unique FP needs, health system factors such as the commodity supply chain, provider level factors such as competence and attitude that influence the quality of care, and structural factors such as infrastructure, equipment and commodities [18–21]. Deficiencies in structural inputs can hinder the delivery of integrated services resulting in missed opportunities to address unmet needs. Evaluating the availability of structural inputs and processes of care necessary for integrated service delivery can help identify barriers to the implementation of integrated FP and HIV services.

Many studies have examined the integration of FP services with HIV programs in one or more health facilities in a single country [14, 22–25]. However, few studies have examined the integration of FP services at a national-level or across multiple countries systematically [20, 21]. Consequently, differences in the availability of integrated FP services in HIV programs across multiple countries have been underexplored. The current study aimed to assess the on-site availability of FP services at HIV care and support sites in 10 sub-Saharan African countries. We also evaluated facility-level factors associated with onsite availability of integrated FP services. Findings from this multi-country analysis reveal critical shortcomings in the implementation of integrated FP and HIV service delivery, and provide evidence to guide the implementation of effective integrated FP and HIV services at scale.

## Methods

### Data sources

Data were obtained from the Service Provision Assessments (SPA) and Service Availability and Readiness Assessments (SARA) [26, 27]. The SPA and SARA are nationally representative, cross-sectional facility-based surveys that collect comprehensive information on the availability and functionality of health systems in the provision of essential health services, including antenatal care, obstetric care, HIV/AIDS care and treatment, and FP. Both surveys include a facility checklist administered to the facility in-charge. The availability of basic amenities, equipment, diagnostics, medicines and commodities for the provision of health services is directly observed and verified by the interviewer. Further details about the survey design and sampling strategy can be found in final survey reports [26, 27].

### Study setting

Our analysis included available data from SPAs and SARAs conducted between 2012 and 2015 in 10 sub-Saharan African countries: Benin, Burkina Faso, Democratic Republic of Congo (DRC), Malawi, Senegal, Sierra Leone, Tanzania, Togo, Uganda, and Zimbabwe. The included countries represent a diversity of populations with a range of sexual and reproductive health needs, and settings with varying nature of the HIV epidemic (Table 1). Overall, contraceptive prevalence rate ranges from 16.2% in Burkina Faso to 66.8% in Zimbabwe, and unmet need for FP ranges from 10.4% in Zimbabwe to 34.3% in Uganda. Coverage of antenatal care (ANC) and facility deliveries is substantial, with between 34 and 76% of pregnant women attending at least four ANC visits, and between 54 and 91% of pregnant women delivering in a health facility. The incidence rate of HIV ranges from 0.08 to 3.03 per 1000 person-years. The burden of HIV and coverage of HIV interventions tends to be higher in countries in the Southern and Eastern Africa regions like Tanzania and Zimbabwe, compared to countries in the Western and Central African regions like the Togo and Sierra Leone. National health expenditures per capita for 2015 ranged from US$20 to US$170.

**Table 1:** State of sexual and reproductive health and the HIV epidemic in 10 sub-Saharan African countries, 2012–2015

	Health expenditure per capita in US^ba^	People living with HIV^b^	HIV incidence §	ART (%)^b^	PMTCT^b^	ANC4+ (%)^c^	Facility deliveries (%)^c^	TFR^c^	Unmet need for FP^c^	CPR (%)^d^
Benin	31	67,000	0.34	57	> 95	59	87	4.9	32.6	17.9
Burkina Faso	33	95,000	0.19	60	83	34	66	6.0	24.5	16.2
DRC	20	370,000	0.17	42	70	48	80	6.6	27.7	20.4
Malawi	34	1,000,000	2.29	66	84	51	91	4.4	18.7	59.2
Senegal	36	41,000	0.08	52	55	47	75	5.0	25.6	23.3
Sierra Leone	107	67,000	–	26	87	76	54	4.9	25.0	16.6
Tanzania	32	1,400,000	1.19	62	84	51	63	5.2	22.1	38.4
Togo	37	100,000	0.59	51	86	57	73	4.8	33.6	19.9
Uganda	46	1,400,000	1.50	67	> 95	48	57	6.2	34.3	30.0
Zimbabwe	94	1,300,000	3.03	75	93	76	77	4.0	10.4	66.8

- indicates no data available. *DRC* Democratic Republic of Congo. *ANC4+*: Coverage of at least four antenatal care visits. *ART:*

*CPR* Contraceptive prevalence rate. *TFR* Total fertility rate. *PMTCT* Antiretroviral therapy coverage for the prevention of mother-to-child HIV transmission

^a^ Source: World Health Organization Global Health Expenditure Database for 2015

^b^ Source: UNAIDS estimates for 2016

^c^ Source: most recent Demographic and Health Surveys or Multiple Indicator Cluster Survey

^d^ Source: United Nations, Department of Economic and Social Affairs, Population Division (2017). World Contraceptive Use 2017 (POP/DB/CP/Rev2017)

### Measures

The study outcome was the availability of on-site integrated FP services at the facility level, which was based on six ‘structural’ and one ‘process of care’ inputs. ‘Structure’ refers to characteristics of the health system in which care is being delivered, while ‘process of care’ describes the care delivered to patients [28]. In line with World Health Organization definition of FP readiness, the structural inputs examined were guidelines on FP, blood pressure apparatus, oral contraceptive pills, injectables, condoms, and trained staff (at least one staff member received training in FP in the previous 1–3 years) [29]. The ‘process of care’ input was the routine provision of FP counselling to HIV/AIDS clients as reported by the facility-in-charge.

Facilities with all the structural and process of care inputs were classified as having onsite integrated FP services; those that did not have one or more inputs were classified as not having onsite integrated FP services. This classification was selected based on the data available in both the SPA and SARA, and did not include other aspects related to the provision of integrated services, such as quality of counselling, patient satisfaction, and provider competence. While the SPAs include other data collections tools (client-provider observations, client exit interviews and health worker interviews) that might reflect a broader range of processes of care, the analysis was limited to the selected inputs to allow the inclusion of several countries with a recent SARA (*n* = 7).

The analysis was restricted to health facilities offering ‘HIV care and support’ services defined as any service directed towards improving the life of a person living with HIV, including treatment of opportunistic infections, provision of palliative care and nutritional rehabilitation. Facilities offering HIV care and support services may offer other HIV services and ancillary health services, which may influence the availability of onsite integrated FP services. Therefore, we examined facility-level measures of the availability of other HIV services (PMTCT, HIV testing and counselling, and HIV/AIDS antiretroviral prescription and client management) and ancillary services (antenatal care, child immunizations, obstetric and newborn care, diagnosis and treatment for sexually transmitted infections, and basic surgical services). Other facility-level characteristics assessed included type of health facility (tertiary versus secondary and primary level), location (urban versus rural), and managing authority (public versus private).

### Analysis

Using descriptive statistics, we summarized the availability of FP services, and structural and process of care inputs across the 10 sub-Saharan African countries. Bivariate and multivariate logistic regression analyses were conducted with availability of onsite integrated FP services as the outcome; backwards stepwise logistic regression was used to determine the facility-level factors associated with the outcome. Analyses included fixed effects for each country. To account for the dependence between facilities within the same country, we used generalized estimating equations to obtain robust standard errors [30]. Associations were presented as odds ratios (OR) with 95% confidence intervals (95% CI), and *p* < 0.05 was considered statistically significant. Multicollinearity among independent variables was assessed using variance inflation factors (VIF). All statistical analyses were appropriately weighted for sample design and performed using STATA 14.2 (College Station, Texas).

## Results

**Fig. 1:**
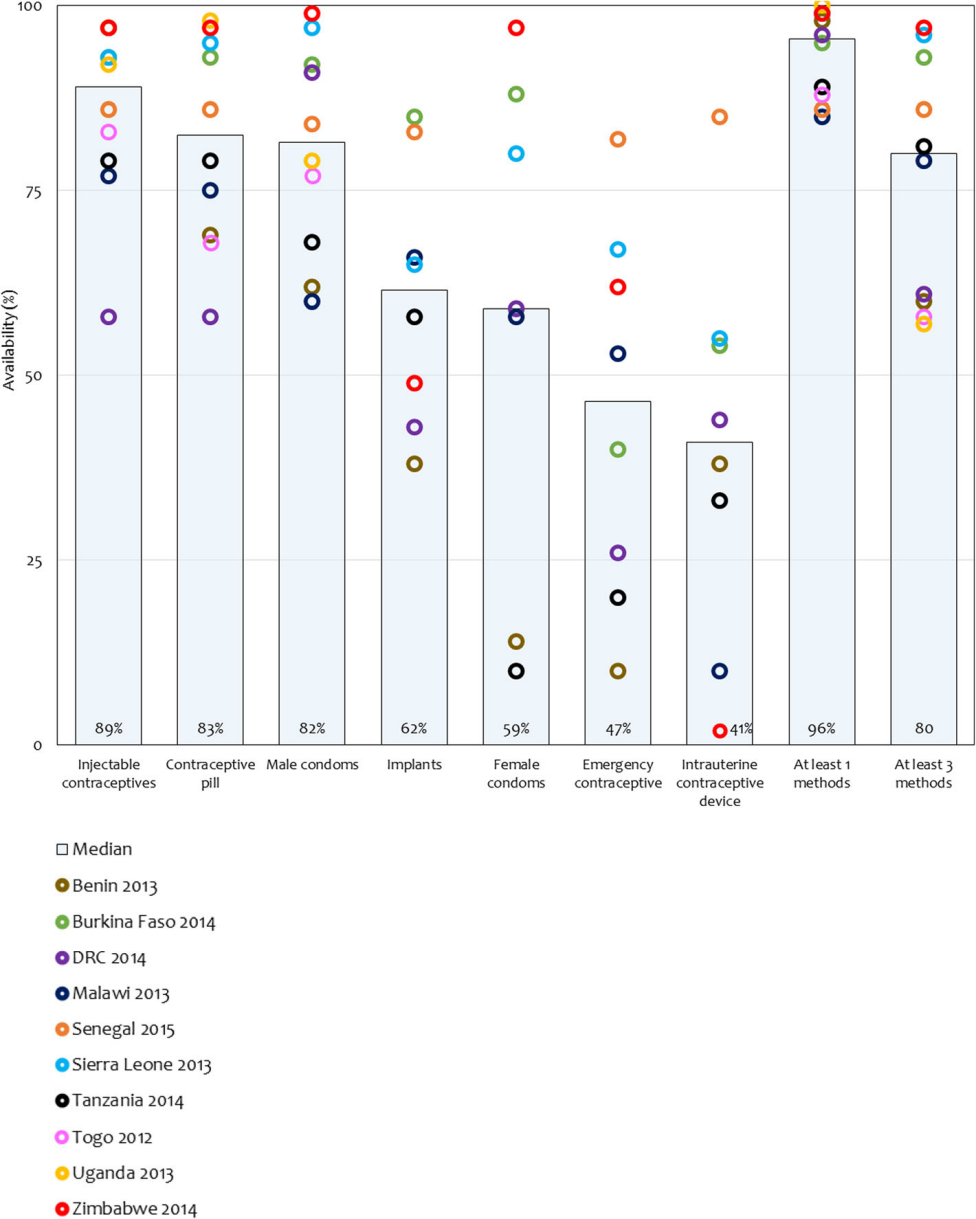
Contraceptive methods available at HIV care and support sites in 10 sub-Saharan African countries

Of the 6209 sampled health facilities, 3161 (51%) offered HIV care and support services and were included in our analytical sample (Table 2). FP services were offered at most HIV care and support sites (across country median: 93%). As an enabling environment is critical to the provision of integrated FP services, we assessed the availability of structural inputs in HIV care and support sites. Whereas blood pressure equipment was widely available (median: 92%), guidelines on FP and staff trained in FP were less available (median: 65 and 50% respectively). By and large, injectable contraceptives, oral contraceptive pills, and male condoms were largely available (median: 89, 83 and 82% respectively). However, there was a relatively lower supply of implants and intrauterine devices (IUDs), which offer long-acting reversible contraception (median: 62 and 41% respectively; Fig. 1). The availability of female condoms and emergency contraception varied widely across countries (range: 10–97% and 10–82%, respectively). Across countries, a median of 95% of the HIV care and support sites had one or more contraceptive methods in stock (range: 85–100%), and 80% had three or more contraceptive methods in stock (range: 57–97%, Fig. 1). Most HIV care and support sites reported routinely providing FP counselling to HIV/AIDS clients – an indicator of the process of care (median: 94%).

**Table 2:** Availability of family planning services in HIV care and support facilities in 10 sub-Saharan African countries, 2012–2015

				Among facilities offering HIV/AIDS care and support, facilities with								
	Health facility survey	Facilities sampled	Facilities offering HIV/AIDS care and support (%)	Offering family planning services (%)	Guidelines on FP (%)	Staff trained in FP (%)	Blood pressure apparatus (%)	Oral contraceptive pills (%)	Injectable contraceptives (%)	Male condoms (%)	Routine provision of family planning counselling to HIV/AIDS clients (%)	Onsite availability of integrated FP services (%)
Benin	SARA (2013)	189	21	98	63	49	86	69	97	62	63	15
Burkina Faso	SARA (2014)	766	88	96	84	75	98	93	93	92	96	61
DRC	SARA (2014)	1555	8	66	47	50	94	58	58	91	83	26
Malawi	SPA (2013)	977	67	85	33	49	87	75	77	60	91	10
Senegal	SPA (2012–14)	483	6	92	74	74	100	86	86	84	95	51
Sierra Leone	SARA (2013)	455	30	94	67	81	91	95	93	97	99	52
Tanzania	SPA (2014–15)	1200	35	89	56	42	92	79	79	68	92	15
Togo	SARA (2012)	100	34	88	70	41	86	68	83	77	86	29
Uganda	SARA (2012)	209	54	93	47	53	86	98	92	79	95	27
Zimbabwe	SARA (2014)	275	98	97	67	49	96	97	97	99	100	37
Median			35	93	65	50	92	83	89	82	94	29

DRC: Democratic Republic of Congo

Based on the availability of structural inputs and process of care indicators, the onsite availability of integrated FP services varied between 10 and 61%, with a median of 29% across all countries (Table 2). Notably, countries with similar onsite availability of integrated FP services showed varying availability of HIV care and support services. Whereas about 26% of HIV care and support sites in DRC and Uganda were classified as having onsite integrated FP services available, only 8% of all sampled facilities offered HIV care and support services in DRC compared to 54% in Uganda.

In the bivariate analyses, the availability of integrated FP services at HIV care and support sites was associated with several facility-level characteristics (Table 3). Except for HIV testing and counselling and diagnosis and treatment of sexually transmitted infection, offering another HIV or ancillary health services was independently associated with higher odds of having integrated FP services. In the multivariate analyses, public HIV care and support sites had higher odds of having onsite integrated FP services available compared with private sites (aOR 1.95, 95% CI: 1.12–3.40; Table 3). Tertiary level sites had higher odds of having integrated FP services available than secondary and primary level sites, though this was only marginally statistically significant (aOR 1.49; 95% CI 1.00–2.22). There was no association between the availability of onsite integrated FP services and rural-urban location (aOR 0.95, 95% CI 0.59–1.54). The odds of having integrated FP services available was higher among HIV care and support sites that provided services for PMTCT (aOR: 3.66, 95% CI 1.10–12.17), antenatal care (aOR: 2.95, 95% CI 1.22–7.14) and basic surgery (aOR: 1.88, 95% CI 1.03–3.43), compared to sites that did not provide these services. With the exception of Togo and Sierra Leone, the odds of having integrated FP services available were significantly higher in Burkina Faso than the remaining countries.

**Table 3:** Univariate and Multivariate analyses of factors associated with onsite availability of integrated family planning services in HIV care and support sites in 10 sub-Saharan African countries, 2012–2015

	Univariate		Multivariate	
Facility-level characteristic	OR	95% CI	aOR	95% CI
Urban	1.32	0.92–1.90	0.95	0.59–1.54
Tertiary level	1.28	0.95–1.73	1.49	1.00–2.22
Public	2.43	1.55–3.83	1.95	1.12–3.40
Offers other HIV ancillary health services				
PMTCT services	10.36	4.22–25.44	3.66	1.10–12.17
Antenatal care	9.20	5.66–14.96	2.95	1.22–7.14
Basic surgery	2.37	1.52–3.69	1.88	1.03–3.43
Obstetric and newborn care	6.89	3.03–15.65	–	
Child immunization	3.47	2.34–5.14	–	
HIV testing and counselling	3.36	0.73–15.47	–	
HIV/AIDS antiretroviral prescriptions	2.73	1.60–4.63	–	
Sexually transmitted infections	0.44	0.10–1.86	–	
Country				
Burkina Faso	–		Reference	
Benin	–		0.13	0.05–0.37
Democratic Republic of Congo	–		0.23	0.14–0.40
Malawi	–		0.09	0.06–0.12
Senegal	–		0.53	0.27–1.05
Sierra Leone	–		0.75	0.50–1.13
Tanzania	–		0.11	0.08–0.16
Togo	–		0.42	0.18–1.03
Uganda	–		0.35	0.19–0.64
Zimbabwe	–		0.59	0.37–0.95

*CI* confidence interval, *OR* odds ratio, *aOR* adjusted odds ratio

## Discussion

Women living with HIV face disproportionately higher rates of unintended pregnancies and the integration of FP and HIV services is a strategy to meet FP needs, reduce the risk of unintended pregnancies, and prevent mother-to-child HIV transmission. This study evaluated the availability of integrated FP services and associated factors in HIV care and support sites across 10 sub-Saharan African countries between 2012 and 2015. Whereas the majority (93%) of HIV care and support sites reported offering FP services in the same location, only 29% of these sites were classified as having onsite integrated FP services available based on the availability of structural and process of care inputs. FP commodities and blood pressure equipment were widely available; however, the availability of guidelines on FP and trained staff were limited. Of note, there were no rural-urban differences in the onsite availability integrated FP services (aOR 0.95, 95% CI 0.59–1.54). This finding is indicative that global efforts to ensure the widespread availability of such services may be gaining traction in both rural and urban settings in sub-Saharan Africa [31]. That said, the lack of trained staff is concerning given the indicator for trained staff, defined as at least one staff member had been trained in any aspect of FP in the previous 1–3 years, represents a minimum requirement. Given the chronic shortage of all cadres of health personnel in the sub-Saharan Africa region and the current rhetoric of double-duty actions, efforts for dual training and supervision of providers should be considered [32, 33]. Barriers to the provision of integrated FP services specifically training and supervision must be addressed if the comprehensive needs of people living with HIV are to be met.

We documented variation across countries in the availability of integrated FP services in HIV care and support sites. Differences in the burden of HIV, FP needs and other health system factors may drive the heterogeneity in the availability of HIV/FP integration. Furthermore, the strengthens and limitations of one model integration over another in different contexts has not been fully explored [34].

Our findings also suggest that HIV care and support sites offering other HIV or ancillary services, specifically PMTCT, ANC and basic surgery services were more likely to have integrated FP services available. Notably, offering PMTCT services was associated with a 3.6-fold increase in the likelihood of onsite integrated FP services, suggesting that HIV care and support sites already equipped with PMTCT units were also inclined to offer integrated FP, or vice versa. Several studies have also documented the positive spillover effect of HIV programs on broader health systems, including the provision and quality of ANC, obstetric care, and child immunization services [35–37]. For instance, one study of health facilities in Kenya found that the presence of PMTCT programs was associated with increased quality of prenatal and postnatal care, specifically the availability of structural inputs [37]. Together, these studies and our findings suggest that investments in supplies, equipment, diagnostics, human resources, medicines and commodities to support the provision of PMTCT services may have substantial indirect benefits on health systems.

We also found that onsite integrated FP services were more available in HIV care and support sites that were government operated and provided tertiary level care. These findings are consistent with studies that have documented supply side deficiencies in the provision of essential health services across sub-Saharan African countries, particularly lower level facilities [36, 38, 39]. In light of efforts to decentralize HIV programs to lower level facilities to ensure improved access and support rapid scale up [40, 41], our results underscore the need for further investments in lower level facilities where a substantial number of patients are expected to receive HIV care and FP in the near future.

There are several limitations worth noting. First, the analysis was based on a secondary analysis of data collected through health facility surveys. Health facility surveys such as the SPA and SARA provide nationally representative information on the state of the health system at one moment in time, and the surveys used reflect service provision between 2012 and 2015. As some HIV care and support sites may provide integrated FP services to few or many HIV clients, without adjustment for care-seeking patterns and health facility caseloads, our data cannot identify the proportion of HIV clients receiving integrated FP services in HIV care and support settings, nor specify the actual content and quality of care received by these clients. The availability of structural inputs and processes of care is a requirement but not a guarantee of provision of integrated FP services. Nevertheless, these findings are still useful in characterizing the environment in which integrated FP and HIV services are being provided and are relevant for national-level policy and program planning.

Second, the present analysis was restricted to assessing the availability of FP services in the same site to HIV-infected women in HIV care and support sites. Due to the nature of the data, it is unclear whether the FP services were provided by the same health provider, or another health provider in the same facility through formal or informal referral. The study did not consider other models of service delivery (e.g. referral based approaches) or the availability of other reproductive services (e.g. those targeted for men and adolescent boys). Data available in the SPA and SARA do not permit the assessment of all integrated service delivery mechanisms operating in HIV care and support sites or other HIV programs like HIV counselling and testing, PMTCT and ART. However, other HIV programs are likely to face parallel challenges in the delivery of integrated FP and HIV services. Further research is needed to better understand the broader spectrum of activities in which HIV care and support sites are engaged to meet the FP needs of both male and female HIV clients.

Third, the definition of availability of onsite integrated FP services was based on the availability of structural inputs and process of care. While the availability of structural inputs was based on direct observation and verification, the indicator for process of care – routine provision of FP counselling to HIV clients was based on report by facility staff, which is prone to misreporting. Also, the definition only considered one indicator of the process of care and may be too crude a measure to represent all dimensions of integrated FP service delivery. Several other indicators have been proposed to track the integration between FP and HIV services at the health facility level with more depth and breadth [42–44]. In addition to service readiness, these evaluation frameworks also consider demand for services, provider training, knowledge and competence, client reports of service provision, and monitoring and evaluation. The use of standard indicators and data collected from client exit interviews and client observations would facilitate the monitoring and evaluation of integrated service delivery and quality. Nevertheless, the definition used in the present study for onsite availability of integrated services reflects the minimum level of service readiness required to provide integrated FP services, which makes it the more concerning that few health facilities met these requirements.

Despite these limitations, our study included health facilities representing a diverse array of settings and contexts, including private, lower level and rural health facilities in 10 sub-Saharan African countries. The included countries represent varying trends in HIV transmission and sexual and reproductive health needs, increasing the generalizability our findings. Together, the 10 countries represent about 23% of the people living with HIV in sub-Saharan Africa.

## Conclusions

Our findings demonstrate that the availability of on-site integrated HIV care and FP services in sub-Saharan Africa is low, despite the provision of FP services being one of the four pillars of the global effort to prevent mother-to-child transmission. There are deficits in the components necessary to provide integrated services, particularly the training of providers. Our findings call for the expansion of integrated FP services to privately owned facilities, lower level facilities and facilities lacking a range of other HIV and ancillary services. The results of this study provide evidence to inform Ministries of Health and other stakeholders in the region on efforts to implement integrated FP and HIV services at scale.

A French translation of this article has been included as Additional file 1.

A Portuguese translation of the abstract has been included as Additional file 2.

## Additional files

Additional file 1: Translation of this article into French. (PDF 674 kb)

Additional file 2: Translation of the abstract of this article into Portuguese. (PDF 176 kb)

## Abbreviations

AIDS: Acquired immunodeficiency syndrome
ANC: Antenatal care
aOR: Adjusted odds ratio
ART: Antiretroviral therapy
CI: Confidence interval
DHS: Demographic and Health Surveys
DRC: Democratic Republic of Congo
FP: Family planning
HIV: Human immunodeficiency virus
IUD: Intrauterine device
OR: Odds ratio
PMTCT: Prevention of mother-to-child HIV transmission
SARA: Service Availability and Readiness Assessment
SPA: Service Provision Assessment
