# Supplementary material for: Availability of integrated family planning services in HIV care and support sites in sub-Saharan Africa: a secondary analysis of national health facility surveys
Source: Reprod Health. 2019 May 29;16(Suppl 1):60. doi: 10.1186/s12978-019-0713-x (PMC6538552; doi:10.1186/s12978-019-0713-x)
Supplement: Supplementary file 1 — Translation of this article into French. (PDF 674 kb) [file 12978_2019_713_MOESM1_ESM.pdf]

# Disponibilité des services de planning familial intégrés sur les sites de soins et de soutien en matière de VIH en Afrique subsaharienne : une analyse secondaire d'enquêtes nationales sur les établissements de santé

Mufaro Kanyangarara[1*], Kwame Sakyi[2], Amos Laar[3]

[1]Department of International Health, Johns Hopkins Bloomberg School of Public Health, Baltimore, Maryland, USA
[2] Department of Public and Environmental Wellness, School of Health Sciences, Oakland University, Rochester, Michigan, USA
[3] Department of Population, Family, and Reproductive Health, School of Public Health, College of Health Sciences, University of Ghana, Accra, Ghana.

*Auteur correspondant: Mufaro Kanyangarara, Department of International Health, Johns Hopkins Bloomberg School of Public Health, 615 N. Wolfe Street, Baltimore, MD, USA. Telephone: 410-502-4561. Email: mkanyan1@jhu.edu

KS: ksakyi@oakland.edu
AL: alaar@ug.edu.gh

## Résumé

**Contexte :** L'intégration du planning familial (PF) aux programmes de soins et de traitement du VIH est une stratégie pour étendre la fourniture de services de PF et éviter les grossesses non désirées parmi les femmes porteuses du VIH. Cependant, on en sait peu sur la disponibilité des services de PF au sein des établissements de santé fournissant des services liés au VIH à travers l'Afrique subsaharienne. Dans cette étude, nous avons évalué la disponibilité des services de PF intégrés et les facteurs associés sur les sites de soins et de soutien en matière de VIH à travers l'Afrique subsaharienne.

**Méthodes :** Nous avons réalisé une analyse secondaire de données des établissements, représentatives à l'échelon national, en provenance des Évaluations de la disponibilité et de la capacité opérationnelle des services (SARA) et des Évaluations de prestations de services (SPA), conduites dans 10 pays de l'Afrique subsaharienne entre 2012 et 2015. Nous avons appliqué six indicateurs reflétant la structure et le processus de soins essentiels à la délivrance de services

de PF dans les établissements de soins et de soutien en matière de VIH, afin de définir le résultat d'intérêt ; à savoir la disponibilité sur place des services de PF intégrés. La régression logistique multivariée a été utilisée pour explorer les caractéristiques associées au résultat au niveau des établissements.

**Résultats :** Parmi les 3 161 établissements de santé offrant des services de soins et de soutien liés au VIH, la plupart ont déclaré offrir également des services de PF sur le même lieu. La disponibilité de trois méthodes de PF était supérieure à la disponibilité des directives de PF et d'un personnel formé. La disponibilité sur place de services de PF intégrés allait de 10 % à 61 %. Les résultats de la régression logistique multivariée indiquent que la probabilité de disposer de services de PF sur place est supérieure sur les sites de soins et de soutien en matière de VIH exploités par le gouvernement, classés comme établissements de soins de niveau tertiaire, et offrant des services de PTME, des soins prénatals et une chirurgie de base.

**Conclusions :** Notre enquête pointe des lacunes critiques dans le degré de préparation des sites de soins et de soutien liés au VIH à délivrer des services de PF intégrés sur place. Des efforts renouvelés sont nécessaires pour traiter ces barrières du côté de l'offre et garantir que les services intégrés de PF et en matière de VIH répondent aux besoins uniques des patients porteurs du VIH.

***Mots clés :*** *VIH, planning familial, Afrique subsaharienne, enquêtes sur les établissements de soins*

## Contexte

Depuis le début de l'épidémie de VIH, on estime que 78 millions de personnes ont été infectées par le virus et que 35 millions sont décédées de causes liées au SIDA [1]. En 2016, 36,7 millions

de personnes vivaient avec le VIH/SIDA dans le monde, dont 25,5 millions en Afrique subsaharienne. Tous les ans, environ 2,1 millions de personnes contractent le VIH, dont 150 000 enfants (<15 ans), subissant pour la plupart une infection verticale via leur mère durant la grossesse, l'accouchement ou l'allaitement [1,2].

L'amplification des interventions pour la prévention de la transmission mère-enfant du VIH (PTME) et des traitements antirétroviraux pour les femmes enceintes et les enfants éligibles a permis de réduire significativement les taux de transmission du VIH, de morbidité et de mortalité [3]. Ces bénéfices associés à un meilleur accès aux traitements à base d'antirétroviraux (TAR) peuvent être contrebalancés par des taux élevés de grossesses non désirées et des niveaux d'utilisation bas de contraceptifs parmi les femmes porteuses du VIH en Afrique subsaharienne [4–8]. La prévention des grossesses non désirées chez les femmes porteuses du VIH est considérée comme un composant essentiel d'une réponse exhaustive au VIH/SIDA, en particulier pour la PTME au niveau mondial [9,10]. L'intégration de services de planning familial (PF) dans les programmes de soins et de traitement liés au VIH/SIDA constitue une approche efficace, à la fois pour réduire la transmission verticale du VIH, pour accroître l'accès à la contraception et pour réduire le taux de mortalité maternelle [11–13]. En outre, au niveau des systèmes de santé, la fourniture de services de PF comme élément d'un continuum de soins liés au VIH peut améliorer l'accès aux services de soins essentiels, l'efficacité des ressources limitées et la pratique clinique [14–16]. Les modèles d'intégration des services en matière de VIH et PF vont de la fourniture de services de PF complets par le même prestataire de soins que les services liés au VIH à des approches basées sur des orientations [17]. L'efficacité d'un modèle particulier d'intégration dépend de facteurs contextuels, tels que la

prévalence du VIH et l'existence de besoins uniques de PF, de facteurs liés au système de santé comme la chaîne d'approvisionnement en produits de base, de facteurs au niveau des prestataires comme les compétences et attitudes qui influencent la qualité des soins, et de facteurs structurels comme les infrastructures, les équipements et produits de base [18–21]. Des défaillances structurelles peuvent faire obstacle à la fourniture de services intégrés et entraîner des occasions manquées de répondre à des besoins non couverts. Évaluer la disponibilité des éléments structurels et des processus de soins nécessaires à l'intégration de services peut contribuer à l'identification des barrières empêchant l'implémentation de services intégrés en matière de PF et de VIH.

De nombreuses études ont examiné l'intégration de services de PF dans des programmes de prise en charge du VIH dans un ou plusieurs établissements de santé d'un même pays [14,22–25]. Toutefois, peu d'études ont analysé de manière systématique l'intégration de services de PF à l'échelon national ou à travers plusieurs pays [20,21]. Par conséquent, les différences de disponibilité des services de PF intégrés dans les programmes liés au VIH à travers plusieurs pays restent mal explorées. La présente étude visait à évaluer la disponibilité sur place des services de PF sur les sites de soins et de soutien en matière de VIH dans 10 pays de l'Afrique subsaharienne. Nous avons également évalué des facteurs au niveau des établissements associés à la disponibilité sur place des services intégrés de PF. Les résultats de cette analyse sur des pays multiples révèlent des lacunes critiques dans la mise en œuvre de la fourniture de services intégrés en matière de PF et VIH et donnent une orientation sur l'implémentation à grande échelle de services intégrés en matière de PF et de VIH.

## Méthodes

### *Sources de données*

Les données proviennent des Évaluations de prestations de services (SPA) et des Évaluations de la disponibilité et de la capacité opérationnelle des services (SARA) [26,27]. Les SPA et SARA sont des enquêtes transversales basées sur les établissements, représentatives des pays, qui collectent des informations exhaustives sur la disponibilité et la fonctionnalité des systèmes de santé dans la délivrance de services de santé, y compris les soins prénatals, les soins obstétriques, les soins et les traitements liés au VIH/SIDA et le PF. Ces deux enquêtes incluent une check-list administrée à l'établissement en charge. La disponibilité des commodités de base, des équipements, des diagnostics, des médicaments et des produits de base pour la fourniture de services de santé est observée directement et vérifiée par l'enquêteur. Des rapports d'enquête finals contiennent plus de détails sur la conception de l'enquête et la stratégie d'échantillonnage [26,27].

### *Configuration de l'étude*

Notre analyse a inclus les données disponibles de SPA et SARA conduites entre 2012 et 2015 dans 10 pays d'Afrique subsaharienne : Bénin, Burkina Faso, République démocratique du Congo. (RDC), Malawi, Sénégal, Sierra Leone, Tanzanie, Togo, Ouganda et Zimbabwe. Les pays inclus couvrent une grande diversité de populations, présentant différents besoins en termes de santé sexuelle et reproductive, et des configurations de nature variée quant à l'épidémie de VIH (Tableau 1). Globalement, le taux de prévalence des contraceptifs va de 16,2 % au Burkina Faso à 66,8 % au Zimbabwe, et les besoins non couverts en PF vont de 10,4 % au Zimbabwe à 34,3 % en Ouganda. La couverture des soins prénatals (SPN) et des naissances en établissement

est substantielle, avec 34 à 76 % de femmes enceintes présentes à au moins quatre visites de SPN, et 54 à 91 % de femmes enceintes accouchant dans un établissement de santé. Le taux d'incidence du VIH va de 0,08 à 3,03 pour 1 000 personnes/an. La prévalence du VIH et la couverture des interventions liées au VIH tendent à être supérieures dans les pays de l'Afrique australe et de l'est, comme la Tanzanie et le Zimbabwe, comparée aux pays d'Afrique de l'ouest et centrale, comme le Togo et la Sierra Leone. Les dépenses nationales de santé par tête pour 2015 allaient de 20 USD à 170 USD.

***Mesures***

L'étude a permis de connaître la disponibilité des services de PF intégrés sur place au niveau d'un établissement, sur la base de six éléments « structurels » et d'un « processus de soins ». « Structurels » se rapporte à des caractéristiques du système de santé dans lequel le soin est délivré, tandis que « processus de soins » décrit le soin délivré aux patients [28]. En accord avec la définition du PF qu'en a fait l'Organisation mondiale de la Santé, les éléments structurels examinés étaient les directives de PF, les tensiomètres, les pilules contraceptives, les contraceptifs injectables, les préservatifs et le personnel formé (au moins un membre du personnel ayant suivi une formation au PF au cours des 1 à 3 années précédentes) [29]. Le « processus de soins » correspondait à la fourniture de routine d'un conseil en PF aux patients touchés par le VIH/sida, tel que déclarée par l'établissement en charge.

Les établissements proposant tous les éléments structurels et de processus de soins étaient classés comme offrant des services de PF intégrés ; ceux qui ne proposaient pas l'un ou l'autre des éléments étaient classés comme n'offrant pas de services de PF intégrés. La classification était choisie sur la base des données disponibles dans les SPA et les SARA, et elle

n'incluait aucun autre aspect lié à la fourniture des services intégrés, tels que la qualité du conseil, la satisfaction des patients et la compétence des prestataires de soins. Alors que les SPA incluent d'autres outils de collecte des données (observations patients/prestataires de soins, entretien de sortie avec les patients et entretiens avec les agents de santé) qui peuvent refléter un éventail plus large de processus de soins, l'analyse s'est limitée aux éléments sélectionnés pour permettre l'inclusion de plusieurs pays disposant d'une SARA récente (n=7).

L'analyse a été restreinte aux établissements de santé proposant des services de « soins et de soutien en matière de VIH », définis comme tout service visant à améliorer la vie d'une personne porteuse du VIH, y compris le traitement des infections opportunistes, la délivrance de soins palliatifs et la réhabilitation nutritionnelle. Les établissements proposant des services de soins et de soutien en matière de VIH peuvent proposer des services de santé auxiliaires qui peuvent influencer la disponibilité de services de PF intégrés sur place. Par conséquent, nous avons examiné les mesures au niveau des établissements de la disponibilité d'autres services liés au VIH (PTME, dépistage du VIH et conseil, prescription d'antirétroviraux pour le VIH/SIDA et gestion des patients) et de services auxiliaires (soins prénatals, immunisations des enfants, soins obstétriques et de néonatalogie, diagnostic et traitement des infections sexuellement transmissibles et services chirurgicaux de base). D'autres caractéristiques évaluées au niveau des établissements incluaient le type d'établissements de santé (niveau tertiaire versus secondaire et primaire), la localisation (urbaine versus rurale) et l'autorité gestionnaire (publique versus privée).

### *Analyse*

Au moyen des statistiques descriptives, nous avons résumé la disponibilité des services de PF et des éléments structurels et de processus de soins à travers les 10 pays d'Afrique subsaharienne. Les analyses à régression logistique bivariée et multivariée ont été conduites avec pour résultat la disponibilité des services de PF sur place ; la régression logistique pas à pas a été utilisée pour déterminer les facteurs associés aux résultats au niveau des établissements. Les analyses incluaient les effets fixes pour chaque pays. Pour expliquer la dépendance entre les établissements d'un même pays, nous avons utilisé des équations d'estimation généralisées pour obtenir des écarts-types robustes [30]. Les associations ont été présentées comme des rapports de cotes (RC) avec des intervalles de confiance de 95 % (IC 95 %), et $p<0,05$ était considéré comme statistiquement significatif. La multicolinéarité parmi des variables indépendantes a été évaluée sur la base de facteurs d'inflation de la variance (FIV). Toutes les analyses statistiques ont été pondérées de manière adaptée au plan d'échantillonnage et ont été réalisées avec le logiciel STATA 14.2 (College Station, Texas).

## Résultats

Sur les 6 209 établissements de santé sondés, 3 161 (51 %) proposaient des services de soins et de soutien en matière de VIH et ont été inclus dans notre échantillon d'analyse (Tableau 2). Des services de PF étaient proposés sur la plupart des sites de soins et de soutien en matière de VIH (médiane sur tous les pays : 93 %). Comme un environnement favorable est critique pour la délivrance de services de PF intégrés, nous avons évalué les disponibilités d'éléments structurels sur les sites de soins et de soutien en matière de VIH. Alors que les tensiomètres étaient largement disponibles (médiane : 92 %), les directives sur le PF et la

présence de personnel formé au PF l'étaient moins (médiane : respectivement 65 % et 50 %). Dans l'ensemble, les contraceptifs injectables, les pilules contraceptives et les préservatifs masculins étaient largement disponibles (médiane : respectivement 89 %, 83 % et 82 %). Cependant, la disponibilité des implants et dispositifs intra-utérins (DIU), qui offrent une contraception de longue durée réversible, était relativement faible (médiane : respectivement 62 % et 41 % ; Figure 1). La disponibilité des préservatifs féminins et de la contraception d'urgence variait sensiblement d'un pays à l'autre (plage : respectivement 10-97 % et 10-82 %). Sur tous les pays, une médiane de 95 % des sites de soins et de soutien en matière de VIH proposaient une ou plusieurs méthodes contraceptives (plage : 85-100 %), et 80 % proposaient trois méthodes contraceptives ou plus (plage : 57-97 %, Figure 1). La plupart des sites de soins et de soutien en matière de VIH ont déclaré fournir des conseils de PF de manière habituelle aux patients atteints du VIH/SIDA ; un indicateur du processus de soins (médiane : 94 %).

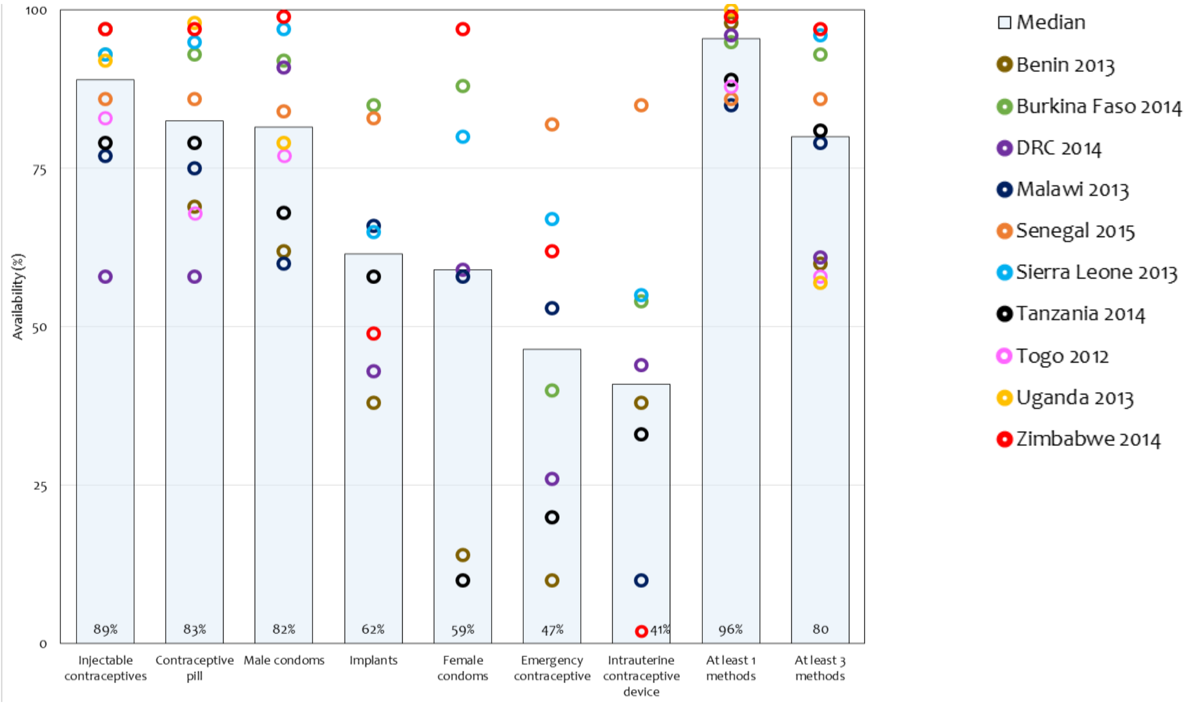


*Figure 1 : les méthodes contraceptives disponibles sur les sites de soins et de soutien en matière de VIH dans 10 pays de l'Afrique subsaharienne*

Sur la base de la disponibilité d'indicateurs d'éléments structurels et de processus de soins, la disponibilité sur place de services de PF intégrés variait de 10 % à 61 %, avec une médiane de 29 % sur la totalité des pays (Tableau 2). Il faut noter que des pays avec une disponibilité sur place similaire de services de PF intégrés ont pourtant une disponibilité variable des services de soins et de soutien en matière de VIH. Alors qu'environ 26 % des sites de soins et de soutien en matière de VIH en RDC et en Ouganda étaient classés disposant de services de PF intégrés sur place, seuls 8 % de tous les établissements sondés offraient des services de soins et de soutien en matière de VIH en RDC, comparé à 54 % en Ouganda.

Dans les analyses bivariées, la disponibilité de services de PF intégrés sur les sites de soins et de soutien en matière de VIH était associée à plusieurs caractéristiques au niveau des établissements (Tableau 3). Hormis pour le dépistage du VIH et le conseil, ainsi que pour le diagnostic et le traitement des infections sexuellement transmissibles, proposer d'autres services de santé liés au VIH ou des services auxiliaires était indépendamment associé à de plus fortes probabilités de proposer des services de PF intégrés. Dans les analyses multivariées, les sites publics de soins et de soutien en matière de VIH avaient plus de probabilités de proposer des services de PF intégrés sur place, comparé aux sites privés (RCc 1,95, IC 95 % : 1,12-3,40 ; Tableau 3). Les sites de niveau tertiaire avaient plus de chances de disposer de services de PF intégrés que les sites de niveau secondaire ou primaire, bien que cela reste statistiquement marginal (RCc 1,49 ; IC 95 % 1,00 – 2,22). Il n'y avait pas de lien entre la disponibilité de services de PF intégrés sur place et la localisation rurale ou urbaine (RCc 0,95, IC 95 % 0,59 – 1,54). Les chances de disposer de services de PF intégrés étaient plus élevées parmi les sites de soins et de soutien en matière de VIH qui proposaient des services de PTME (RCc : 3,66, IC 95 % 1,10 –

12,17), des soins prénatals (RCc : 2,95, IC 95 % 1,22 – 7,14) et une chirurgie de base (RCc : 1,88, IC 95 % 1,03 – 3,43), comparé aux sites qui ne proposaient pas ces services. À l'exception du Togo et de la Sierra Leone, les chances de disposer de services de PF intégrés étaient significativement plus élevées au Burkina Faso que dans les autres pays.

**Discussion**

Les femmes porteuses du VIH présentent des taux disproportionnellement élevés de grossesses non désirées et l'intégration de services de PF et liés au VIH constitue une stratégie pour répondre aux besoins de PF, réduire le risque de grossesse non désirée et éviter la transmission mère-enfant du VIH. Cette étude a évalué la disponibilité des services de PF intégrés et les facteurs associés sur les sites de soins et de soutien en matière de VIH à travers 10 pays de l'Afrique subsaharienne entre 2012 et 2015. Alors que la majorité (93 %) des sites de soins et de soutien en matière de VIH déclarait offrir des services de PF sur place, seuls 29 % de ces sites ont été classés comme proposant des services de PF intégrés, sur la base d'éléments structurels et de processus de soins. Les produits de base du PF et les tensiomètres étaient largement disponibles ; cependant, la disponibilité de directives de PF et de personnel formé était limitée. À noter qu'il n'y avait pas de différence « rural/urbain » dans la disponibilité de services de PF intégrés (RCc 0,95, IC 95 % 0,59 – 1,54). Ceci indique que les efforts globaux pour garantir la disponibilité étendue de ces services font effet dans les localités rurales et urbaines de l'Afrique subsaharienne [31]. Ceci dit, le manque de personnel formé est inquiétant vu que l'indicateur pour le personnel formé, défini comme au moins un membre du personnel ayant suivi une formation sur un quelconque aspect du PF au cours des 1 à 3 années précédentes, constitue une exigence minimale. Étant donné le manque chronique de cadres de santé dans la

région de l'Afrique subsaharienne et la rhétorique actuelle sur les actions à double obligation, des efforts de formation double et la supervision des prestataires de soins devraient être considérés [32,33]. Les obstacles à la fourniture de services de PF intégrés, en particulier la formation et la supervision, doivent être traités si l'on souhaite couvrir tous les besoins des personnes porteuses du VIH.

Nous avons documenté les variations de disponibilité des services de PF intégrés sur les sites de soins et de soutien en matière de VIH à travers les différents pays. Les différences en termes de prévalence du VIH, de besoins en PF et d'autres facteurs des systèmes de santé peuvent causer des disponibilités hétérogènes pour l'intégration en matière de VIH/PF. En outre, les forces et les limites d'un modèle d'intégration sur un autre dans différents contextes n'ont pas été pleinement explorées [34].

Nos résultats suggèrent aussi que les sites de soins et de soutien en matière de VIH offrant d'autres services liés au VIH ou des services auxiliaires, en particulier de PTME, de SPN et de chirurgie de base, étaient plus susceptibles de disposer de services de PF intégrés. On remarque qu'offrir des services de PTME était associé à une multiplication par 3,6 de la probabilité d'avoir des services de PF sur place, ce qui suggère que les sites de soins et de soutien en matière de VIH déjà équipés d'unités de PTME étaient également enclins à proposer un PF intégré, et vice versa. Plusieurs études ont également documenté l'effet d'entraînement positif des programmes liés au VIH sur des systèmes de santé plus larges, y compris la fourniture et la qualité des services de SPN, de soins obstétriques et de vaccination des enfants [35–37]. Par exemple, une étude sur des établissements de santé au Kenya indique que la présence de programmes de PTME était associée à une meilleure qualité des soins prénatals et

postnatals, en particulier la disponibilité d'éléments structurels [37]. Au total, ces études et nos résultats suggèrent qu'investir dans les fournitures, les équipements, les diagnostics, les ressources humaines, les médicaments et les produits de base, pour appuyer la délivrance de services de PTME, peut avoir des bénéfices substantiels indirects sur les systèmes de santé.

Nous avons également découvert que les services de PF intégrés sur place étaient plus disponibles sur les sites gérés par l'État et proposant des soins de niveau tertiaire. Ces résultats sont cohérents avec des études qui ont documenté les défaillances du côté de l'offre dans la fourniture de services de santé essentiels à travers des pays de l'Afrique subsaharienne, en particulier avec des établissements de niveau de soins inférieur [36,38,39]. Compte tenu des efforts de décentralisation des programmes liés au VIH vers des établissements de niveau de soins inférieurs pour assurer un meilleur accès et soutenir une amplification rapide [40,41], nos résultats soulignent le besoin d'investissements accrus dans les établissements de niveau de soins inférieurs où l'on attend un nombre important de patients nécessitant des soins liés au VIH et au PF dans un proche avenir.

Il y a plusieurs limitations à noter. Premièrement, l'analyse était basée sur une analyse secondaire de données collectées via des enquêtes sur des établissements de santé. Les enquêtes sur les établissements de santé, telles que les SPA et SARA, fournissent des informations représentatives sur l'état du système de santé un moment donné, et les enquêtes utilisaient la fourniture de services reflétée entre 2012 et 2015. Comme certains sites de soins et de soutien en matière de VIH peuvent proposer des services de PF a peu ou à beaucoup de patients séropositifs, sans ajustement des schémas de recherche de soins et des charges de travail des établissements de santé, nos données ne permettent pas d'identifier la proportion

de patients séropositifs recevant des services PF intégrés sur les lieux de soins et de soutien en matière de VIH, ni de préciser le contenu réel et la qualité des soins reçus par ces patients. La disponibilité des éléments structurels et des processus de soins est une exigence mais pas une garantie pour la fourniture de services de PF intégrés. Néanmoins, ces résultats sont utiles pour caractériser l'environnement dans lequel les services liés au PF et au VIH sont fournis et sont pertinents pour la planification des politiques et des programmes au niveau national.

Deuxièmement, la présente analyse était restreinte à l'évaluation de la disponibilité de services de PF sur un même lieu, pour les femmes porteuses du VIH sur les sites de soins et de soutien en matière de VIH. En raison de la nature des données, on ne sait pas si les services de PF étaient proposés par le même prestataire de soins ou par un autre prestataire du même établissement, via une procédure d'orientation formelle ou informelle. L'étude n'a pas considéré d'autres modèles de prestations de services (p. ex. approches basées sur une orientation) ou la disponibilité d'autres services liés à la reproduction (p. ex. ceux ciblant les hommes et les adolescents garçons). Les données disponibles dans les études SPA et SARA ne permettent pas d'évaluer tous les mécanismes de fourniture de services intégrés opérant sur les sites de soins et de soutien en matière de VIH, ou d'autres programmes liés au VIH, tels que le conseil et le dépistage, la PTME et les TAR. Cependant, d'autres programmes liés au VIH sont susceptibles d'affronter des défis similaires dans la fourniture de services intégrés en matière de PF et de VIH. Il faut plus de recherches pour mieux comprendre tout l'éventail d'activités dans lequel les sites de soins et de soutien en matière de VIH sont engagés pour répondre aux besoins de PF des patients séropositifs, hommes et femmes.

Troisièmement, la définition de la disponibilité sur place de services de PF intégrés était basée sur la disponibilité d'éléments structurels et du processus de soins. Alors que la disponibilité d'éléments structurels était basée sur l'observation directe et la vérification, l'indicateur du processus de soins – dispense systématique de conseils liés au PF pour les patients séropositifs - était basé sur les déclarations du personnel de l'établissement, sujettes à falsification. De même, la définition considérait un seul indicateur du processus de soins et peut s'avérer être une mesure trop brute pour représenter toutes les dimensions de la fourniture de services de PF intégrés. Plusieurs autres indicateurs ont été proposés pour suivre l'intégration entre les services liés au PF et au VIH au niveau des établissements de santé, avec une approche plus étendue et plus profonde [42–44]. En plus de la disponibilité des services, ces cadres d'évaluation considèrent également la demande de services, de formation, de connaissances et de compétences des prestataires de soins, de rapport des patients sur la fourniture de services, et de surveillance et d'évaluation. L'utilisation d'indicateurs standard et de données collectées lors d'entretiens de sortie avec les patients et d'observations des patients faciliterait la surveillance et l'évaluation de la fourniture et de la qualité des services intégrés. Néanmoins, la définition utilisée dans la présente étude concernant la disponibilité sur place de services intégrés reflète le niveau minimum de services requis pour proposer des services de PF intégrés, ce qui rend encore plus inquiétant le fait que peu d'établissements de santé aient été conformes à ces exigences.

Malgré ces limitations, notre étude incluait des établissements de santé représentant une grande diversité de lieux et de contextes, avec des établissements de santé privés, de niveau de soins inférieurs et ruraux, dans 10 pays de l'Afrique subsaharienne. Les pays inclus

illustrant des tendances variables dans les besoins liés à la transmission du VIH et à la santé sexuelle et reproductive, ce qui accroît la généralisabilité de nos résultats. Au total, les 10 pays représentent environ 23 % des personnes séropositives vivant en Afrique subsaharienne.

## Conclusions

Nos résultats démontrent que la disponibilité sur place des services intégrés en matière de VIH et PF en Afrique subsaharienne est faible, bien que la fourniture de services de PF constitue l'un des quatre piliers de l'effort global de prévention de la transmission mère-enfant. Il y a des déficits au niveau des composantes nécessaires pour proposer des services intégrés, en particulier dans la formation des prestataires de soins. Nos résultats plaident pour l'extension de services de PF intégrés aux établissements du secteur privé, aux établissements de niveau de soins inférieur et aux établissements qui ne proposent pas un éventail de services liés au VIH et de services auxiliaires. Cette étude démontre aux ministères de la santé et autres parties prenantes dans la région que des efforts s'imposent pour la mise en œuvre à grande échelle de services intégrés en matière de PF et de VIH.

## Liste des abréviations

SIDA : syndrome de l'immunodéficience acquise
SPN : soins prénatals
RCc : rapports de cotes corrigés
TAR : traitement antirétroviral
IC : intervalle de confiance
EDS : enquêtes démographiques et de santé
RDC : République démocratique du Congo
PF : planification familiale
VIH : virus de l'immunodéficience humaine
DIU : dispositif intra-utérin
RC : rapports de cotes
PTME : prévention de la transmission mère-enfant du VIH
SARA : Service Availability and Readiness Assessment (évaluation de la disponibilité et de la capacité opérationnelle des services)
SPA : Service Provision Assessment (évaluation des prestations de services)

**Concernant ce supplément**

Cet article a été publié comme partie de l'ouvrage *Reproductive Health*, Volume 16 Supplement 1, 2019: Effective Integration of Sexual Reproductive Health and HIV Prevention, Treatment, and Care Services across sub-Saharan Africa: Where is the evidence for program implementation?
Le supplément a été publié dans le cadre d'une collaboration entre *Reproductive Health* et *BMC Public Health.* L'intégralité du contenu, avec les versions en français, en portugais et en anglais, est disponible en ligne :
https://bmcpublichealth.biomedcentral.com/articles/supplements/volume-19-supplement-1
et
https://reproductive-health-journal.biomedcentral.com/articles/supplements/volume-16-supplement-1

**Déclarations**

**Conflits d'intérêts**

Les auteurs déclarent ne pas avoir de conflits d'intérêts.

**Approbation éthique et accord de participation**

Non applicable.

**Accord de publication**

Non applicable.

**Disponibilité des données et matériels**

Toutes les données utilisées dans ce manuscrit peuvent être demandées à l'adresse :

https://dhsprogram.com/Data/ et

http://www.who.int/healthinfo/systems/sara_introduction/en/.

**Financement**

Le supplément de la revue est rendu possible grâce au soutien généreux du peuple américain via la United States Agency for International Development (USAID) en partenariat avec le Fonds des Nations unies pour la population (FNUAP) et le Programme commun des Nations Unies sur le VIH/SIDA (ONUSIDA).

Les opinions exprimées dans la présente publication sont celles des auteurs et ne reflètent pas nécessairement les politiques officielles de l'USAID, du FNUAP ou de l'ONUSIDA, la mention des dénominations de ministères ou d'organismes n'implique pas non plus l'aval du gouvernement américain, du FNUAP ou de l'ONUSIDA.

**Contributions des auteurs**

MK, AL et KS ont conjointement conçu cette étude. MK a conduit l'analyse et rédigé la première ébauche du papier. AL et KS ont fourni un examen et des commentaires critiques. Tous les auteurs ont lu et approuvé le manuscrit final.

**Références**

1. Joint United Nations Programme on HIV/AIDS. UNAIDS DATA 2017. 2017.

2. De Cock KM, Fowler MG, Mercier E, de Vincenzi I, Saba J, Hoff E, et al. Prevention of mother-to-child HIV transmission in resource-poor countries: translating research into policy and practice. JAMA. 2000 Mar;283(9):1175–82.

3. UNAIDS, UNICEF, World Health Organization. Global HIV/AIDS response: epidemic update and health sector progress towards universal access: progress report 2011. World Health Organization; 2011.

4. Schwartz SR, Rees H, Mehta S, Venter WDF, Taha TE, Black V. High incidence of unplanned pregnancy after antiretroviral therapy initiation: findings from a prospective cohort study in South Africa. PLoS One. 2012;7(4):e36039.

5. Adair T. Unmet need for contraception among HIV-positive women in Lesotho and implications for mother-to-child transmission. J Biosoc Sci. 2009 Mar;41(2):269–78.

6. Warren CE, Abuya T, Askew I. Family planning practices and pregnancy intentions among HIV-positive and HIV-negative postpartum women in Swaziland: a cross sectional survey. BMC Pregnancy Childbirth. 2013 Jul;13:150.

7. McCoy SI, Buzdugan R, Ralph LJ, Mushavi A, Mahomva A, Hakobyan A, et al. Unmet need for family planning, contraceptive failure, and unintended pregnancy among HIV-infected and HIV-uninfected women in Zimbabwe. PLoS One. 2014;9(8):e105320.

8. Laar AK, Taylor AE, Akasoe BA. HIV-seropositivity is not important in childbearing decision-making among HIV-positive Ghanaian women receiving antiretroviral therapy. AIDS Care. 2015;27(7):870–5.

9. World Health Organization. The Glion Call to Action on family planning and HIV/AIDS in women and children. WHO Geneva. 2004;

10. World Health Organization. World Malaria Report 2014. 2014.

11. Tsui AO, McDonald-Mosley R, Burke AE. Family Planning and the Burden of Unintended Pregnancies. Epidemiol Rev. 2010;32(1):152–74.

12. Reynolds HW, Janowitz B, Homan R, Johnson L. The value of contraception to prevent perinatal HIV transmission. Sex Transm Dis. 2006 Jun;33(6):350–6.

13. Stover J, Fuchs N, Halperin D, Gibbons A, Gillespie D. Adding family planning to PMTCT sites increases the benefits of PMTCT. Issue Br. 2003;

14. Grossman D, Onono M, Newmann SJ, Blat C, Bukusi EA, Shade SB, et al. Integration of family planning services into HIV care and treatment in Kenya: a cluster-randomized trial. AIDS. 2013 Oct;27 Suppl 1:S77-85.

15. Church K, Mayhew SH. Integration of STI and HIV prevention, care, and treatment into family planning services: a review of the literature. Stud Fam Plann. 2009 Sep;40(3):171–86.

16. Shade SB, Kevany S, Onono M, Ochieng G, Steinfeld RL, Grossman D, et al. Cost, cost-efficiency and cost-effectiveness of integrated family planning and HIV services. AIDS. 2013 Oct;27 Suppl 1:S87-92.

17. Haberlen SA, Narasimhan M, Beres LK, Kennedy CE. Integration of Family Planning Services into HIV Care and Treatment Services: A Systematic Review. Stud Fam Plann. 2017;48(2):153–77.

18. Hope R, Kendall T, Langer A, Bärnighausen T. Health Systems Integration of Sexual and Reproductive Health and HIV Services in Sub-Saharan Africa: A Scoping Study. Vol. 67, Journal of Acquired Immune Deficiency Syndromes (1999). 2014. p. S259-70.

19. Fajans P, Huntington D. Integrating sexual and reproductive health-care services: Policy Brief. Geneva; 2006.

20. Adamchak S, Janowitz B, Liku J, Munyambanza E, Grey T, Keyes E. Study of family planning and HIV integrated services in five countries. Fam Heal Int Res Triangle Park NC, USA. 2010;

21. Johnson K, Varallyay I, Ametepi P. Integration of HIV and family planning health services in sub-Saharan Africa: A review of the literature current recommendations and evidence from the Service Provision Assessment Health Facility Surveys. 2012;

22. Kosgei RJ, Lubano KM, Shen C, Wools-Kaloustian KK, Musick BS, Siika AM, et al. Impact of integrated family planning and HIV care services on contraceptive use and pregnancy outcomes: a retrospective cohort study. J Acquir Immune Defic Syndr. 2011 Dec;58(5):e121-6.

23. Phiri S, Feldacker C, Chaweza T, Mlundira L, Tweya H, Speight C, et al. Integrating reproductive health services into HIV care: strategies for successful implementation in a low-resource HIV clinic in Lilongwe, Malawi. BMJ Sex Reprod Heal. 2016;42(1):17–23.

24. Haddad LB, Feldacker C, Jamieson DJ, Tweya H, Cwiak C, Bryant AG, et al. Medical eligibility, contraceptive choice, and intrauterine device acceptance among HIV-infected women receiving antiretroviral therapy in Lilongwe, Malawi. Int J Gynaecol Obstet. 2014 Sep;126(3):213–6.

25. Haddad LB, Feldacker C, Jamieson DJ, Tweya H, Cwiak C, Chaweza T, et al. Pregnancy Prevention and Condom Use Practices among HIV-Infected Women on Antiretroviral Therapy Seeking Family Planning in Lilongwe, Malawi. PLoS One. 2015;10(3):1–14.

26. The DHS Program - Service Provision Assessments (SPA) [Internet]. [cited 2016 Oct 31]. Available from: http://dhsprogram.com/What-We-Do/Survey-Types/SPA.cfm

27. WHO | Service availability and readiness assessment (SARA) [Internet]. [cited 2017 Oct

31]. Available from: http://www.who.int/healthinfo/systems/sara_introduction/en/

28. Donabedian A. The quality of care: how can it be assessed? Jama. 1988;260(12):1743–8.

29. World Health Organization. Service availability and readiness assessment (SARA): an annual monitoring system for service delivery: reference manual. 2013;

30. Lipsitz SR, Laird NM, Harrington DP. Generalized estimating equations for correlated binary data: using the odds ratio as a measure of association. Biometrika. 1991;78(1):153–60.

31. Bongaarts J, Cleland J, Townsend JW, Bertrand JT, Gupta M Das. Family planning programs for the 21st century. New York Popul Counc. 2012;

32. Bharat S, Mahendra VS. Meeting the sexual and reproductive health needs of people living with HIV: challenges for health care providers. Reprod Health Matters. 2007 May;15(29 Suppl):93–112.

33. Kane R, Wellings K. Staff training in integrated sexual health services. Sex Transm Infect. 2003;79(5):354–6.

34. Mayhew SH, Hopkins J, Warren CE. Building integrated health systems: lessons from HIV, sexual and reproductive health integration. Health Policy Plan. 2017;32(suppl_4):iv1-iv5.

35. Ekouevi DK, Stringer E, Coetzee D, Tih P, Creek T, Stinson K, et al. Health facility characteristics and their relationship to coverage of PMTCT of HIV services across four African countries: The PEARL study. PLoS One. 2012;7(1):1–7.

36. Kruk ME, Leslie HH, Verguet S, Mbaruku GM, Adanu RMK, Langer A. Quality of basic

maternal care functions in health facilities of five African countries: an analysis of national health system surveys. Lancet Glob Heal. 2016;(16):1–11.

37. Kruk ME, Jakubowski A, Rabkin M, Kimanga DO, Kundu F, Lim T, et al. Association Between HIV Programs and Quality of Maternal Health Inputs and Processes in Kenya. Am J Public Health. 2015;105(S2):S207–10.

38. Kanyangarara M, Walker N, Munos M. Quality of Antenatal Care Service Provision in Health Facilities across Sub-Saharan Africa: Evidence from Nationally Representative Health Facility Assessments. J Glob Health. 2017;7(2).

39. Church K, Machiyama K, Todd J, Njamwea B, Mwangome M, Hosegood V, et al. Identifying gaps in HIV service delivery across the diagnosis-to-treatment cascade: findings from health facility surveys in six sub-Saharan countries. Vol. 20, Journal of the International AIDS Society. 2017.

40. Fayorsey RN, Saito S, Carter RJ, Gusmao E, Frederix K, Koech-Keter E, et al. Decentralization of Pediatric HIV Care and Treatment in Five Sub-Saharan African Countries. JAIDS J Acquir Immune Defic Syndr. 2013;62(5).

41. Scanlon ML, Vreeman RC. Current strategies for improving access and adherence to antiretroviral therapies in resource-limited settings. Vol. 5, HIV/AIDS (Auckland, N.Z.). 2013. p. 1–17.

42. SRH and HIV Linkages Compendium: Indicators and Related Assessment Tools, prepared and published by IPPF, UNFPA and WHO on behalf of the Interagency Working Group on

SRH and HIV Linkages. London; 2014.

43. Freyder M, Craig L, Kaji A. Monitoring the Integration of Family Planning and HIV Services A Manual to Support the Use of Indicators to Measure Progress toward PEPFAR's 90-90-90 Targets and Protect Women's Reproductive Rights. Chapel Hill, North Carolina; 2016.

44. Adamchak SE, Okello FO, Kabore I. Developing a system to monitor family planning and HIV service integration: results from a pilot test of indicators. J Fam Plan Reprod Heal care. 2016 Jan;42(1):24–9.

## Tableaux

**Tableau 1 : état de la santé sexuelle et reproductive et de l'épidémie de VIH dans 10 pays d'Afrique subsaharienne, 2012-2015**

| | Dépenses de santé par tête en US$* | Personnes porteuses du VIH§ | Incidence du VIH § | TAR (%) § | PTME § | SPN4+ (%) † | Naissances en établissement (%) † | TFT † | Besoins non couverts de PF† | TPC (%) ‡ |
|---|---|---|---|---|---|---|---|---|---|---|
| Bénin | 31 | 67 000 | 0,34 | 57 | >95 | 59 | 87 | 4,9 | 32,6 | 17,9 |
| Burkina Faso | 33 | 95 000 | 0,19 | 60 | 83 | 34 | 66 | 6,0 | 24,5 | 16,2 |
| RDC | 20 | 370 000 | 0,17 | 42 | 70 | 48 | 80 | 6,6 | 27,7 | 20,4 |
| Malawi | 34 | 1 000 000 | 2,29 | 66 | 84 | 51 | 91 | 4,4 | 18,7 | 59,2 |
| Sénégal | 36 | 41 000 | 0,08 | 52 | 55 | 47 | 75 | 5,0 | 25,6 | 23,3 |
| Sierra Leone | 107 | 67 000 | - | 26 | 87 | 76 | 54 | 4,9 | 25,0 | 16,6 |
| Tanzanie | 32 | 1 400 000 | 1,19 | 62 | 84 | 51 | 63 | 5,2 | 22,1 | 38,4 |
| Togo | 37 | 100 000 | 0,59 | 51 | 86 | 57 | 73 | 4,8 | 33,6 | 19,9 |
| Ouganda | 46 | 1 400 000 | 1,50 | 67 | >95 | 48 | 57 | 6,2 | 34,3 | 30,0 |
| Zimbabwe | 94 | 1 300 000 | 3,03 | 75 | 93 | 76 | 77 | 4,0 | 10,4 | 66,8 |

- indique l'absence de données disponibles. RDC : République démocratique du Congo. SPN4+ : au moins quatre visites de soins prénatals couvertes. TAR :

TPC : taux de prévalence des contraceptifs. TFT : taux de fertilité total. PTME : couverture par traitement antirétroviral de la prévention de la transmission mère-enfant du VIH.

* Source : Base de données des dépenses de santé mondiales de l'Organisation mondiale de la Santé pour 2015.

§ Source : estimations de l'ONUSIDA pour 2016.

† Source : dernières enquêtes démographiques et de santé ou enquête par grappe à indicateurs multiples.

‡ Source :

**Tableau 2 : disponibilité des services de planning familial dans les établissements de soins et de soutien en matière de VIH dans 10 pays d'Afrique subsaharienne, 2012 – 2015**

| | | | | Parmi les établissements proposant des soins et un soutien en matière de VIH/SIDA, établissements avec | | | | | | | | |
|---|---|---|---|---|---|---|---|---|---|---|---|---|
| | Enquête sur les établissements de santé | Établissements sondés | Établissements proposant des soins et un soutien en matière de VIH/SIDA (%) | Services de planning familial (%) | Directives sur le PF (%) | Personnel formé au PF (%) | Tensiomètres (%) | Pilules contraceptives (%) | Contraceptifs injectables (%) | Préservatifs masculins (%) | Fourniture en routine de conseils de planning familial aux patients VIH/SIDA (%) | Disponibilité sur place de services intégrés de PF (%) |
| Bénin | SARA (2013) | 189 | 21 | 98 | 63 | 49 | 86 | 69 | 97 | 62 | 63 | 15 |
| Burkina Faso | SARA (2014) | 766 | 88 | 96 | 84 | 75 | 98 | 93 | 93 | 92 | 96 | 61 |
| RDC | SARA (2014) | 1 555 | 8 | 66 | 47 | 50 | 94 | 58 | 58 | 91 | 83 | 26 |
| Malawi | SPA (2013) | 977 | 67 | 85 | 33 | 49 | 87 | 75 | 77 | 60 | 91 | 10 |
| Sénégal | SPA (2012-14) | 483 | 6 | 92 | 74 | 74 | 100 | 86 | 86 | 84 | 95 | 51 |
| Sierra Leone | SARA (2013) | 455 | 30 | 94 | 67 | 81 | 91 | 95 | 93 | 97 | 99 | 52 |
| Tanzanie | SPA (2014-15) | 1 200 | 35 | 89 | 56 | 42 | 92 | 79 | 79 | 68 | 92 | 15 |
| Togo | SARA | 100 | 34 | 88 | 70 | 41 | 86 | 68 | 83 | 77 | 86 | 29 |

| | | | | | | | | | | | | |
|---|---|---|---|---|---|---|---|---|---|---|---|---|
| | (2012) | | | | | | | | | | | |
| Ougan da | SARA (2012) | 209 | 54 | 93 | 47 | 53 | 86 | 98 | 92 | 79 | 95 | 27 |
| Zimbab we | SARA (2014) | 275 | 98 | 97 | 67 | 49 | 96 | 97 | 97 | 99 | 100 | 37 |
| **Média ne** | | | **35** | **93** | **65** | **50** | **92** | **83** | **89** | **82** | **94** | **29** |

RDC : République démocratique du Congo

**Tableau 3 : analyses univariées et multivariées des facteurs associés à la disponibilité sur place de services intégrés de planning familial sur les sites de soins et de soutien en matière de VIH dans 10 pays d'Afrique subsaharienne, 2012-2015**

| | **Univariées** | | **Multivariées** | |
|---|---|---|---|---|
| **Caractéristique au niveau de l'établissement** | RC | IC 95 % | RCc | IC 95 % |
| Urbain | 1,32 | 0,92 – 1,90 | 0,95 | 0,59 – 1,54 |
| Niveau tertiaire | 1,28 | 0,95 – 1,73 | 1,49 | 1,00 – 2,22 |
| Public | 2,43 | 1,55 – 3,83 | 1,95 | 1,12 – 3,40 |
| Propose d'autres services de santé auxiliaires liés au VIH | | | | |
| Services PTME | 10,36 | 4,22 – 25,44 | 3,66 | 1,10 – 12,17 |
| Soins prénatals | 9,20 | 5,66 – 14,96 | 2,95 | 1,22 – 7,14 |
| Chirurgie de base | 2,37 | 1,52 – 3,69 | 1,88 | 1,03 – 3,43 |
| Soins obstétriques et de néonatalogie | 6,89 | 3,03 – 15,65 | - | |
| Immunisation des enfants | 3,47 | 2,34 – 5,14 | - | |
| Tests de dépistage du VIH et conseils | 3,36 | 0,73 – 15,47 | - | |
| Prescriptions d'antirétroviraux pour le VIH/SIDA | 2,73 | 1,60 – 4,63 | - | |
| Infections sexuellement transmissibles | 0,44 | 0,10 – 1,86 | - | |
| Pays | | | | |
| Burkina Faso | - | | Référence | |
| Bénin | - | | 0,13 | 0,05 – 0,37 |
| République démocratique du Congo | - | | 0,23 | 0,14 – 0,40 |
| Malawi | - | | 0,09 | 0,06 – 0,12 |
| Sénégal | - | | 0,53 | 0,27 – 1,05 |
| Sierra Leone | - | | 0,75 | 0,50 – 1,13 |
| Tanzanie | - | | 0,11 | 0,08 – 0,16 |
| Togo | - | | 0,42 | 0,18 – 1,03 |
| Ouganda | - | | 0,35 | 0,19 – 0,64 |
| Zimbabwe | - | | 0,59 | 0,37 – 0,95 |

IC : intervalle de confiance ; RC : rapports de cotes ; RCc : rapports de cotes corrigés
