# Supplementary material for: Availability of integrated family planning services in HIV care and support sites in sub-Saharan Africa: a secondary analysis of national health facility surveys
Source: Reprod Health. 2019 May 29;16(Suppl 1):60. doi: 10.1186/s12978-019-0713-x (PMC6538552; doi:10.1186/s12978-019-0713-x)
Supplement: Supplementary file 2 — Translation of the abstract of this article into Portuguese. (PDF 176 kb) [file 12978_2019_713_MOESM2_ESM.pdf]

Reproductive Health 2019, 16 (Suppl 1) [translated from the original publication]

Disponibilidade de serviços integrados de planeamento familiar em instituições de apoio e cuidados de VIH na África Subsariana: uma análise secundária dos inquéritos nacionais sobre instalações de saúde

Mufaro Kanyangarara[1*], Kwame Sakyi[2], Amos Laar[3]

[1]Department of International Health, Johns Hopkins Bloomberg School of Public Health, Baltimore, Maryland, USA
[2] Department of Public and Environmental Wellness, School of Health Sciences, Oakland University, Rochester, Michigan, USA
[3] Department of Population, Family, and Reproductive Health, School of Public Health, College of Health Sciences, University of Ghana, Accra, Ghana.

* Autor correspondente: Mufaro Kanyangarara, Department of International Health, Johns Hopkins Bloomberg School of Public Health, 615 N. Wolfe Street, Baltimore, MD, USA. Telephone: 410-502-4561. Email: mkanyan1@jhu.edu

KS: ksakyi@oakland.edu
AL: alaar@ug.edu.gh

Resumo

Introdução: Integrar serviços de planeamento familiar (PF) com programas de tratamento e cuidados de VIH é uma estratégia para aumentar a prestação dos serviços de PF e impedir gravidezes indesejadas entre mulheres que vivem com o VIH. Contudo, pouco se sabe sobre a disponibilidade dos serviços de PF nas instalações de saúde que fornecem serviços do VIH em toda a África Subsariana. Neste estudo, a disponibilidade dos serviços integrados de PF foi avaliada, bem como os fatores associados a instituições de apoio e cuidados de VIH em toda a África Subsariana.

Métodos: Foi realizada uma análise secundária dos dados nacionais ao nível das instalações, utilizando os inquéritos Service Availability and Readiness Assessments (SARA) e Service Provision Assessments (SPA) em 10 países da África Subsariana entre 2012 e 2015. Também foram utilizados seis indicadores que refletem a estrutura e o processo dos cuidados essenciais para a prestação dos serviços de PF nas instituições de apoio e cuidados de VIH para definir o resultado de interesse, ou seja, a disponibilidade no local de serviços

integrados de PF. Uma regressão logística multivariada foi utilizada para explorar as características a nível das instalações associadas ao resultado.

Resultados: Entre as 3161 instalações de saúde que fornecem serviços de apoio e cuidados de VIH, a maioria referiu que também oferece serviços de PF no mesmo local. A disponibilidade dos três métodos de PF foi superior à disponibilidade de diretrizes e formação de pessoal no âmbito do PF. A disponibilidade no local de serviços integrados de PF variou entre 10% e 61%. Os resultados da regressão logística multivariada indicam que a probabilidade de ter serviços integrados de PF disponíveis no local foi mais elevada nas instalações de apoio e cuidados de VIH geridas pelo governo, classificadas como um centro de cuidados de nível superior que fornece serviços para PTMF, cuidados pré-natais e cirurgia básica.

Conclusões: Os resultados indicam que existem insuficiências significativas relativamente à preparação das instalações de apoio e cuidados de VIH para fornecer serviços integrados de PF no local. São necessárias novas iniciativas para dar resposta a estes obstáculos ao nível de oferta e assegurar que os serviços integrados de PF e VIH satisfazem as necessidades únicas dos utentes com VIH.

Palavras-chave: VIH, planeamento familiar, África Subsariana, inquéritos sobre instalações de saúde

Sobre este suplemento

Este artigo foi publicado como parte da revista científica *Reproductive Health*, Volume 16, Suplemento 1, 2019: Integração Eficaz dos Serviços de Saúde Sexual e Reprodutiva e de Prevenção, Cuidados e Tratamento do VIH na África Subsariana: Onde estão as provas da implementação do programa?

O suplemento foi publicado como uma colaboração entre as revistas científicas *Reproductive Health* e *BMC Public Health.* O conteúdo integral, incluindo as versões em francês, português e inglês, estão disponíveis online:
https://bmcpublichealth.biomedcentral.com/articles/supplements/volume-19-supplement-1
e
https://reproductive-health-journal.biomedcentral.com/articles/supplements/volume-16-supplement-1
